# Device-Associated Infections in COVID-19 Patients: Frequency of Resistant Bacteria, Predictors and Mortality in Medellín, Colombia

**DOI:** 10.3390/microorganisms12040640

**Published:** 2024-03-22

**Authors:** Diana Patricia Ocampo, Lina María Echeverri-Toro, Judy Natalia Jiménez, Lorena Salazar, Carlos Vargas, Gustavo Roncancio, Maria Alejandra Roa, Johanna Marcela Vanegas

**Affiliations:** 1 Faculty of Medicine, School of Health Sciences, Pontifical Bolivarian University, Medellín 050031, Colombia; diana.ocampo@upb.edu.co (D.P.O.); liecheverri@hptu.org.co (L.M.E.-T.); groncancio@vid.org.co (G.R.); 2 Pablo Tobón Uribe Hospital, Medellín 050010, Colombia; 3 Research Group in Basic and Applied Microbiology, School of Microbiology, University of Antioquia, Medellín 050010, Colombia; jnatalia.jimenez@udea.edu.co (J.N.J.); lorena.salazaro@udea.edu.co (L.S.); 4 School of Health Sciencies, Remington University Corporation, Medellín 050010, Colombia; carlos.vargas01@uniremington.edu.co; 5 CardioVID Clínic, Medellín 050010, Colombia; 6 Secretaría de Salud, Medellín 050010, Colombia; maria.roa@medellin.gov.co

**Keywords:** COVID-19, healthcare-associated infections, device-associated infections, bacterial resistance, carbapenemases

## Abstract

Introduction: Increased antimicrobial use during the COVID-19 pandemic has raised concerns about the spread of resistant bacteria. This study analyzed the frequency of device-associated infections (DAI) caused by resistant bacteria, the predictors of these infections, and 30-day all-cause mortality in patients with and without COVID-19. Methods: A retrospective cohort study was conducted on DAI patients admitted to the ICU (intensive care unit) in 20 hospitals in Medellin, Colombia (2020–2021). The exposure assessed was the COVID-19 diagnosis, and outcomes analyzed were resistant bacterial infections and 30-day mortality. Clinical and microbiological information was collected from surveillance databases. Statistical analysis included generalized linear mixed-effects models. Results: Of the 1521 patients included, 1033 (67.9%) were COVID-19-positive and 1665 DAI were presented. Carbapenem-resistant *Enterobacteriaceae* (CRE) infections predominated during the study (n = 98; 9.9%). The patients with COVID-19 had a higher frequency of metallo-beta-lactamase-producing CRE infections (n = 15; 33.3%) compared to patients without the disease (n = 3; 13.0%). Long-stay in the ICU (RR: 2.09; 95% CI: 1.39–3.16), diabetes (RR: 1.73; 95% CI: 1.21–2.49), and mechanical ventilation (RR: 2.13; 95% CI: 1.01–4.51) were CRE infection predictors in COVID-19 patients, with a mortality rate of 60.3%. Conclusion: CRE infections were predominant in COVID-19 patients. In pandemic situations, the strategies to control DAI should be maintained to avoid infections caused by resistant bacteria, such as length of stay in the ICU and duration of mechanical ventilation.

## 1. Introduction

Most COVID-19 patients present with mild-to-moderate symptoms; however, approximately 20% of patients require hospitalization, and between 5% and 8% develop severe symptoms requiring ICU (intensive care unit) admission [1,2]. This can have adverse effects, such as the occurrence of healthcare-associated infections (HAI), which prolong hospital stays and are associated with high mortality rates [3].

The pandemic has posed significant challenges to healthcare, as the rapid spread of the infection required rapid changes in hospital practices, often neglecting the surveillance, prevention, and control of HAI [4]. Many hospitals were faced with having spaces that were not adequate for ICUs, adding to the need to include new beds due to the increase in serious cases of COVID-19 [5]. Likewise, hospitals had a limited number of specialized personnel for the care of critical patients and insufficient protective equipment for health professionals [6].

On the other hand, during the pandemic, empirical antibiotics were widely used, and antimicrobial stewardship programs were discontinued due to concerns about the prevalence of bacterial coinfections and their high frequency in outbreaks caused by other coronaviruses, such as severe acute respiratory syndrome coronavirus (SARS-CoV) and Middle East respiratory syndrome coronavirus (MERS-CoV), which may have increased bacterial resistance [7]. Although coinfections in patients with COVID-19 can vary, with reported percentages ranging from 1–14%, it is evident that their presence worsens the prognosis, increasing mortality up to fivefold [8,9,10,11].

Thus, different studies have reported the widespread use of antimicrobial therapies as part of the care plan for patients with COVID-19 infection, and it has generally been estimated that up to 90% of patients have received empirical antimicrobial treatment [10,12,13,14,15]. Additionally, a multicenter international study that included data from 23 countries and 82 hospitals in Europe and North America revealed a use of broad-spectrum antimicrobials in patients with COVID-19 of 61.8%, with the decision to use antimicrobial therapy being based more on clinical presentation than on laboratory markers of infection or radiological findings [16]. Furthermore, the World Health Organization states that in the coming years, the trend of resistance will be driven by the incorrect use of antibiotics during the pandemic, as patients with COVID-19 have been empirically treated with antibiotics to prevent subsequent complications due to bacterial coinfections [11].

This indicates that the global situation faced during the COVID-19 pandemic has had a significant impact on the issue of HAI and bacterial resistance; however, the incidence of these infections in COVID-19 patients may be underestimated, especially in developing countries where the number of HAI rates is historically elevated. In Colombia, for example, in September 2020, critical care beds were increased by 91% compared to February of the same year, alongside a high percentage of patients requiring mechanical ventilation, urinary catheters, and intravascular devices [17]. This situation led to a high risk of device-associated infections (DAI), which are the most common healthcare-associated infections in intensive care units. Additionally, the country recorded resistance rates to antibiotics such as third-generation cephalosporins ranging from 25.3% to 34.7% and carbapenems at 15.6% in *K. pneumoniae*, a highly prevalent microorganism isolated from adult ICU patients [18].

In this context, it is vital to understand the behavior of antimicrobial resistance during the COVID-19 pandemic, especially in endemic areas with the circulation of resistant bacteria like Colombia. This study analyzed the frequency of device-associated infections (DAI) caused by resistant bacteria, the factors predicting these infections, and the 30-day all-cause mortality in patients with and without COVID-19 hospitalized in ICUs in Medellín, Colombia.

## 2. Materials and Methods

### 2.1. Setting and Population

This retrospective observational cohort study was carried out in 20 hospitals in Medellín, Colombia. All patients diagnosed with device-associated infections in the ICU between March 2020 and May 2021 were included. The infections evaluated were ventilator-associated pneumonia (VAP), central-line-associated bloodstream infection (CLABSI), and catheter-associated urinary tract infection (CAUTI), according to the Centers for Disease Control and Prevention National Healthcare Safety Network (CDC-NHSN) definitions [19]. Patients under 18 years of age, those with DAI caused by microorganisms other than bacteria, and those with inconsistencies or missing data in the records were excluded. Patients who met the eligibility criteria during the study period were enrolled. Approval for the study was obtained from the Ethics Committee of the School of Health Sciences at Universidad Pontificia Bolivariana (Approval No. 12 of 2021).

### 2.2. Variables

The eligible patients were categorized for analysis into those who had COVID-19 and those who did not. A confirmed diagnosis of COVID-19 was defined as a positive result in a polymerase chain reaction (PCR) or antigen detection test in patients hospitalized in the ICU. Both patients admitted to the hospital with a diagnosis of the disease and those diagnosed during the hospitalization period were included. The primary outcome was the diagnosis of DAI caused by clinically significant antimicrobial-resistant bacteria, such as methicillin-resistant *Staphylococcus aureus* (MRSA), extended-spectrum beta-lactamase-producing *Enterobacteriaceae* (ESBL-E), carbapenem-resistant *Enterobacteriaceae* (CRE), carbapenem-resistant non-fermenting Gram-negative bacilli (CRNF-GNB), and vancomycin-resistant *Enterococcus* spp. (VRE). Additionally, 30-day all-cause mortality after the diagnosis of DAI was evaluated as a secondary outcome. Potential predictors of infections caused by resistant bacteria included age, length of stay in the ICU, and the presence of comorbidities such as diabetes and obesity, as well as exposure to invasive medical devices (urinary catheters, central venous catheters, and mechanical ventilation). 

### 2.3. Data Collection

The information was collected from four databases. Demographic data such as sex, age, place of origin, type of insurance, and clinical information, including comorbidities, as well as the use of antimicrobials in defined daily doses (DDD), were obtained from the database generated from the epidemiological surveillance of healthcare-associated infections (HAI) by the Health Department of Medellín. These data are periodically collected in accordance with the national guidelines and policies of the National Institute of Health within the framework of the prevention, surveillance, and control program for HAI and antimicrobial resistance in Colombia [20]. 

Microbiological information was obtained from a second database constructed with reports from the WHONET surveillance system (developed by the World Health Organization), which are provided monthly by high-complexity hospitals in Medellín to the epidemiological surveillance team. This database facilitates the recognition of the microbiological phenotype of the monitored HAI, the behavior of antimicrobial resistance, the type of sample, and the results of microbiological tests reported, such as the antimicrobial inhibition method with ethylenediaminetetraacetic acid (EDTA) and boronic acid (APB), and the modified carbapenem inactivation method (mCIM). The results of the COVID-19 tests were obtained from a third database with daily reports of cases with positive tests reported by each city to the National Institute of Health. Finally, all-cause mortality during the 30 days after the diagnosis of DAI was obtained from the death records database of the city of Medellín. All the information was consolidated and unified into a single database based on each patient’s identification number.

### 2.4. Statistical Analysis

Qualitative variables were expressed as absolute and relative frequencies and compared using the chi-square test of independence. On the other hand, continuous quantitative variables were presented descriptively using the median and interquartile range, as they did not meet the assumption of normality, which was assessed using the Shapiro–Francia test. These variables were compared using the Mann–Whitney U test. Generalized linear mixed-effects models with Poisson distribution, log link, and variance robust estimator were applied to identify potential predictors of infections caused by antibiotic-resistant bacteria in patients with COVID-19. Measures of association were reported as relative risks (RRs) with 95% confidence intervals and *p*-values. The correlation between the number of deaths from any cause within 30 days after the diagnosis of DAI and the number of COVID-19 cases was descriptively analyzed using scatterplots, considering different antibiotic-resistant bacteria. The analysis was conducted using STATA v.15.

## 3. Results

Of 2108 eligible patients during the study period, 1521 were included (Figure S1, Supplementary Material). The median age was 64 (IQR: 52–71), 954 (62.7%) were male, and the most frequent comorbidities were diabetes (n = 336; 22.1%) and obesity (n = 298; 19.6%). A total of 1033 (67.9%) had a confirmed diagnosis of COVID-19, with a median time from symptom onset to diagnosis of 5 days (IQR: 4–10). The frequency of obesity (22.5% vs. 13.5%) and previous infections (16.7% vs. 8.8%) was higher in patients with COVID-19 compared to those without the disease, respectively (Table S1, Supplementary Material). 

### 3.1. Device-Associated Infections in COVID-19 Patients

One thousand six hundred and sixty-five DAI episodes occurred (144 patients had more than one DAI), and 989 microorganisms were isolated from the study population. The most prevalent DAI was VAP (n = 802; 48.2%), followed by CLABSI (n = 538; 32.3%) and CAUTI (n = 325; 19.5%). The median time from the diagnosis of COVID-19 to the first device-associated infection was 13 days (IQR: 9–19). The frequency of ventilator-associated pneumonia (VAP) was higher in patients with COVID-19 (n = 618; 54.1%), as well as the presence of a central venous catheter (n = 809; 70.8%), mechanical ventilation (n = 1007; 88.1%), and infections caused by *Klebsiella pneumoniae* (n = 195; 32.2%); with statistically significant differences (Table 1). The frequency of each microorganism for each device-associated infection in patients with COVID-19 is presented in Table S2 (Supplementary Material).

### 3.2. Overall Use of Antibiotics and Antimicrobial Resistance

At the beginning of the study, the main antibiotic used in the adult ICU was piperacillin-tazobactam (DDD = 15.1 between March and May 2020); however, its consumption decreased in the following months. Other antimicrobials, such as cefepime and meropenem, were commonly used, reaching DDDs of 12 and 11.9, respectively, between March and May 2021 (Figure S2, Supplementary Material). 

**Figure 1:**
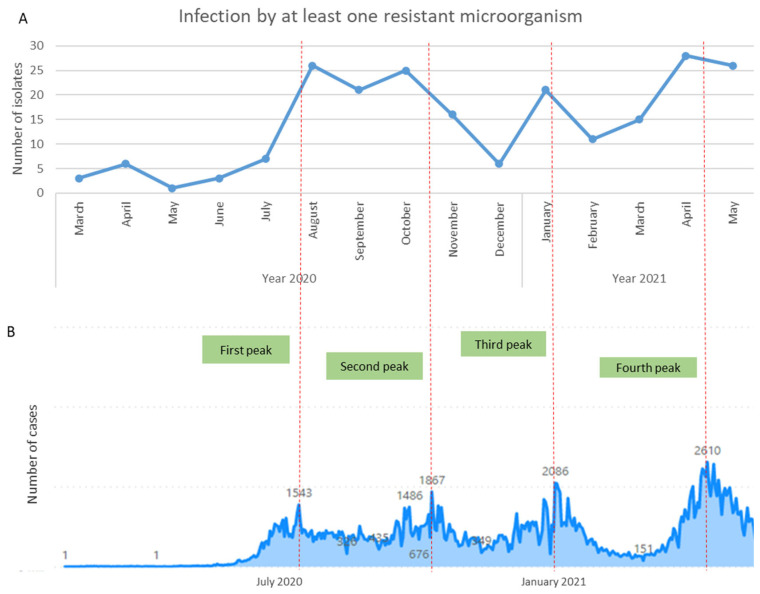
COVID-19 cases and resistant microorganisms causing device-associated infections in Medellín between March 2020 and May 2021. (**A**) Infections caused by at least one resistant microorganism (methicillin-resistant *Staphylococcus aureus*, vancomycin-resistant *Enterococcus* spp., Gram-negative bacteria producing ESBLs, or carbapenem-resistant Gram-negative bacteria). Data from WHONET; (**B**) COVID-19 peaks in Medellín (between July and August 2020, between October and November 2020, January 2021, and between April and May 2021). Data from National Institute of Health, Colombia.

Regarding antimicrobial resistance, it was found that during the peaks of the COVID-19 pandemic in Medellín (between July and August 2020, between October and November 2020, January 2021, and between April and May 2021), there was an increase in DAI caused by at least one resistant microorganism (MRSA, VRE, ESBL-producing *Enterobacteriaceae*, CRE, or non-fermenting carbapenem-resistant Gram-negative bacilli) (Figure 1). 

### 3.3. Infections Caused by Resistant Bacteria and Predictors

**Figure 2:**
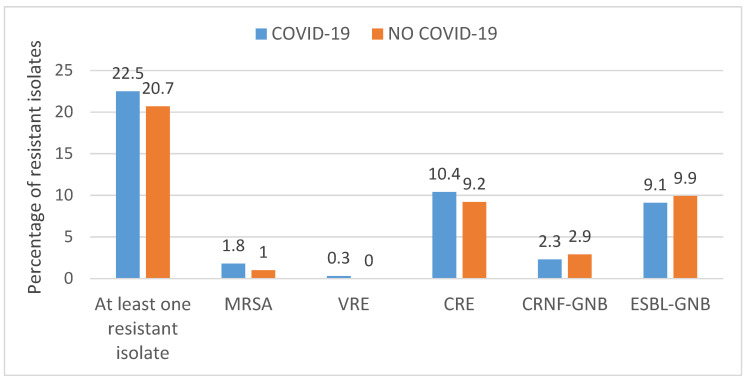
Frequency of resistant microorganisms according to the diagnosis of COVID-19. MRSA: methicillin-resistant *Staphylococcus aureus*; VRE: vancomycin-resistant *Enterococcus* spp.; CRE: carbapenem-resistant *Enterobacteriaceae*; carbapenem-resistant non-fermenting Gram-negative bacilli (CRNF-GNB); and ESBL-producing Gram-negative bacilli (ESBL-GNB).

Among 987 infections, carbapenem-resistant *Enterobacteriaceae* were common (n = 98; 9.9%), followed by ESBL-producing *Enterobacteriaceae* (n = 93; 9.4%), non-fermenting carbapenem-resistant Gram-negative bacilli (n = 25; 2.5%), MRSA (n = 15; 1.5%), and VRE isolates (n = 2; 0.2%). Similar frequencies of infections caused by resistant bacteria were observed in patients with COVID-19 compared to patients without the disease (Figure 2). 

Of the 98 carbapenem-resistant *Enterobacteriaceae* isolates, 69 were tested using the EDTA inhibition method for the detection of carbapenemases, and 18 of the isolates (26.1%) tested positive for serine or metallo-beta-lactamases. Patients with COVID-19 had a higher percentage of infections caused by metallo-beta-lactamase-producing CRE (n = 15; 33.3%) compared to patients without the disease (n = 3; 13.0%). In the case of infections caused by serine-carbapenemase-producing isolates, the percentage was similar in patients with and without COVID-19 (n = 30; 65.2% vs. n = 15; 62.5%).

Regarding the predictors of infections caused by resistant bacteria, in the case of carbapenem-resistant *Enterobacteriaceae*, factors such as an ICU stay longer than 14 days (RR: 2.09; 95% CI: 1.39–3.16), a diagnosis of diabetes (RR: 1.73; 95% CI: 1.21–2.49), and the presence of mechanical ventilation (RR: 2.13; 95%CI: 0.99–4.51) stood out, with statistically significant differences. No differences were observed for infections caused by other resistant bacteria (Table 2).

### 3.4. Mortality and Antimicrobial Resistance

**Figure 3:**
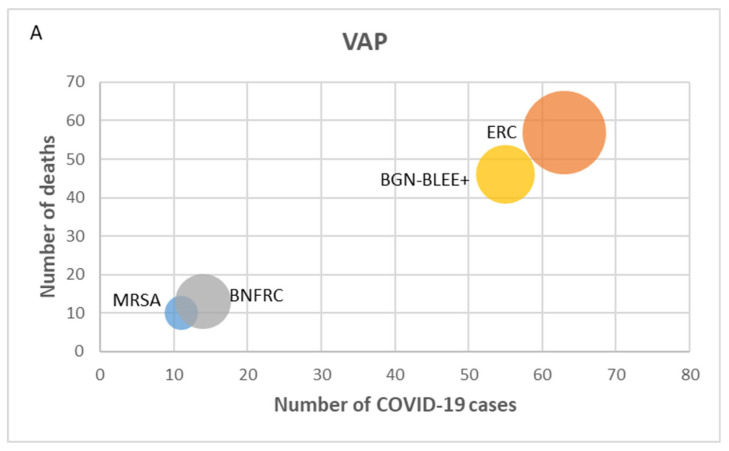

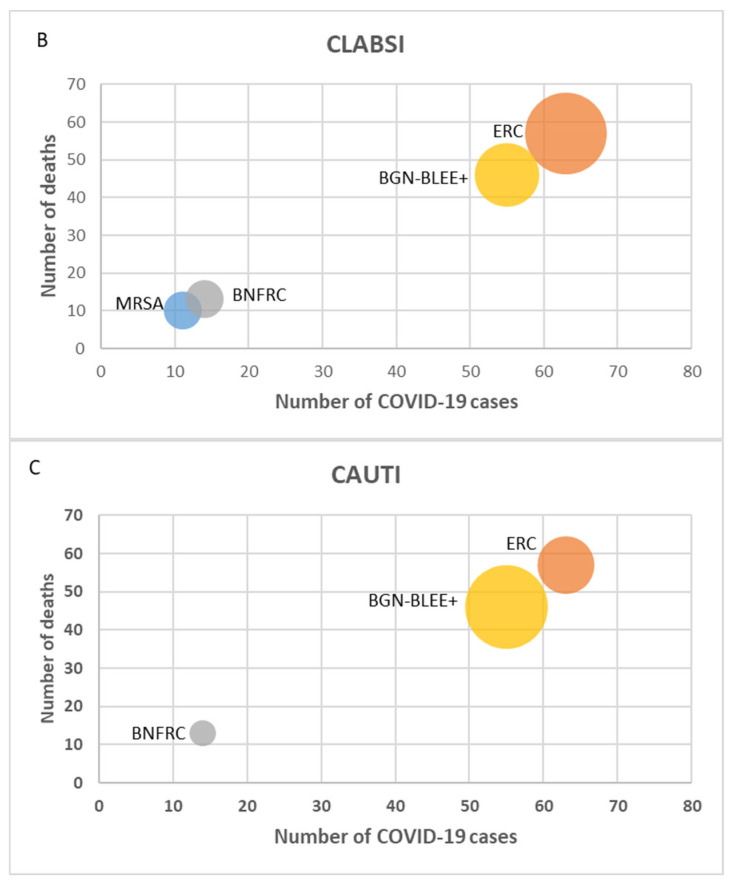
Relationship between COVID-19 cases and mortality. The size of the bubble is proportional to the number of deaths caused by each resistant bacterium. (**A**) VAP: ventilator-associated pneumonia; (**B**) CAUTI: catheter-associated urinary tract infection; (**C**) CLABSI: central-line-associated bloodstream infection. MRSA: methicillin-resistant *Staphylococcus aureus*, VRE: vancomycin-resistant *Enterococcus* spp.; CRE: carbapenem-resistant *Enterobacteriaceae*, carbapenem-resistant non-fermenting Gram-negative bacilli (CRNF-GNB), and ESBL-producing Gram-negative bacilli (ESBL-GNB).

The total number of deaths within 30 days from any cause was 842 (55.4%). For all types of DAI, it was observed that the number of deaths increased with the number of COVID-19 cases. Deaths were more frequent in patients with infections caused by carbapenem-resistant *Enterobacteriaceae* in the cases of VAP and CLABSI (Figure 3). 

## 4. Discussion

In this study, hospitalized ICU patients diagnosed with COVID-19 who developed DAI had a higher frequency of infections caused by metallo-beta-lactamase-producing CRE (33.3%) compared to patients without the infection (13.0%). This finding is not isolated and has been observed on various continents. In Italy, colonization by carbapenem-resistant microorganisms producing metallo-beta-lactamases was reported in over one-third of COVID-19 patients (38.3%) who had spent at least one day in the ICU [21]. In New York, 62.5% of patients with severe COVID-19 had co-infection with New Delhi metallo-beta-lactamase (NDM)-producing *Enterobacteriaceae*, which were isolated from blood or respiratory samples. Similarly, in Mexico, an outbreak of NDM-producing *Escherichia coli* was described in COVID-19 patients, primarily causing pneumonia and UTI, with 71% of patients dead with septic shock [6]. In Colombia, a report from the National Institute of Health about carbapenemase-producing *Enterobacteriaceae* showed an increase in the co-production of beta-lactamases. They rose from 15.2% in 2019 to 59.2% in 2021, with KPC+NDM co-production being the most common. *Klebsiella pneumoniae* was the reported microorganism in 18 out of 50 outbreaks during the pandemic and predominantly exhibited a carbapenem resistance phenotype, which aligns with the findings of this study, as *K. pneumoniae* was the most common in patients with COVID-19 [22]. However, these results contrast with another study conducted in the United States, which reported an increase in the incidence of DAI in 2021 during the surge of COVID-19 cases, but the most frequently documented microorganism was MRSA [23].

Regarding the presence of outbreaks, antimicrobial resistance surveillance data from the National Institute of Health revealed a 257% increase in reported outbreaks in 2021 compared to the year 2020. The predominance of carbapenem-resistant bacteria producing metallo-beta-lactamases in this study may be explained by the occurrence of outbreaks in one or several institutions in the city, as seen in Mexico and Italy. During the pandemic period, the dissemination of these resistant pathogens in the ICU has been associated with increased use of immunosuppressive drugs, empirical antimicrobial use, modification of care protocols regarding the use and reuse of healthcare personnel protection equipment, and decreased care and surveillance of invasive devices in critically ill patients [24]. Additionally, it is possible that healthcare professionals focused primarily on self-protection rather than the prevention of cross-transmission of microorganisms, overcrowded COVID-19 patient care units, and the low number of professionals with adequate infection control training [25,26]. There is evidence that the implemented isolation measures to prevent COVID-19 transmission were not sufficient to contain such outbreaks and epidemiological events associated with clinically significant infections in critically ill patients, which ultimately contributed to the severity and mortality of ICU patients [27].

On the other hand, this study identified mechanical ventilation, diabetes, and prolonged stay in the ICU as predictors of infections caused by carbapenem-resistant enterobacteria, which is consistent with the findings of Falcone et al., who also demonstrated these predictors of superinfections in patients with COVID-19 [26]. Furthermore, it is recognized that viral respiratory infections predispose to secondary bacterial infections, thereby increasing the severity of the disease and mortality [28]. This highlights the importance of establishing strategies to prevent healthcare-associated infections in COVID-19 patients, such as limiting mechanical ventilation to the shortest possible duration and optimizing treatment and diagnostic measures in the ICU to reduce the length of stay. It is crucial to identify factors that predict infections caused by resistant bacteria in order to design diagnostic pathways for timely detection and treatment.

Particularly, the use of antibiotics is a recognized risk factor for the emergence of infections caused by resistant bacteria. Although this study was unable to evaluate individual antibiotic use, the defined daily doses (DDDs) reflected a high consumption of broad-spectrum beta-lactams such as piperacillin-tazobactam, meropenem, and cefepime in the ICUs of the city. Some authors have shown the use of broad-spectrum antibiotics in up to 72% of patients with COVID-19 in critical and non-critical settings [29]. Similar findings were reported in other studies, which demonstrated an increase in the consumption of broad-spectrum antimicrobials such as beta-lactams in combination with beta-lactamase inhibitors, extended-spectrum cephalosporins, carbapenems, and glycopeptides, among others, during the first year of the pandemic [5,30,31].

Regarding the number of deaths, this study showed an increase in mortality among patients with DAI as the number of COVID-19 cases increased, with a higher number of deaths in patients with ERC infections, mainly in the cases of CLABSI and NAV. Infections caused by resistant microorganisms have been associated with increased hospital stays and mortality [32,33,34]. Particularly in the case of ERC infections, in-hospital mortality rates range from 30% to 80% [35]. A multicenter study conducted in Italy reported a high risk of acquiring NAV and CLABSI caused by multidrug-resistant microorganisms in patients with COVID-19, accounting for one-third of all DAI, and although no association between infection and mortality was demonstrated, DAI complicated by septic shock nearly doubled the mortality rate [36].

With respect to the characteristics of patients with COVID-19, most patients in our study were men, with an average age of 64 years and comorbidities such as diabetes and obesity. These findings are consistent with studies conducted in England and Spain, where the most important comorbidities were hypertension, diabetes mellitus, and coronary artery disease [4,37]. This strengthens the evidence that the presence of comorbidities and advanced age increase morbidity and mortality in critically ill patients with COVID-19 [38].

As strengths of this study, it is worth highlighting the inclusion of different hospitals, increasing external validity, and the use of different databases that provide a complete overview of DAI and the behavior of antimicrobial resistance during the first year of the pandemic. The hospitals admit diverse populations from both Medellín and other regions of the country, offering high-complexity healthcare services across a range of medical and surgical specialties.

In terms of limitations, being a retrospective study, it was not possible to conduct a more rigorous and specific analysis of the molecular, microbiological, and clinical characteristics of the isolates causing DAI in patients with COVID-19. The variant of SARS-CoV-2 was not determined; hence, it was not possible to analyze the relationship between the circulating variant of SARS-CoV-2 and device-associated infections.

Additionally, this study could have information biases due to the absence of clinical data such as the use of antibiotics for each patient, current treatment with immunosuppressants, pregnancy, and antibiotic time. The mortality rate was analyzed descriptively without making causal inferences due to the absence of variables that are essential for the comparative analysis. All-cause mortality evaluated in this study did not provide detailed information about the causes of death or if the deaths were due to the infection or not. It is necessary to design and implement studies that allow for timely and real-time evaluation of the consequences of bacterial resistance in critically ill patients during epidemiological emergencies, considering molecular aspects and improved tools for microbiological diagnosis that enable timely treatment and management of DAIs.

In order to mitigate the risk of device contamination and subsequent infections, multifaceted prevention and control strategies are essential. Implementation of evidence-based practices such as strict adherence to aseptic techniques during device insertion and maintenance, regular assessment of the necessity of device use, and prompt removal of devices when no longer needed is paramount. Additionally, healthcare facilities should prioritize continuous education and training for healthcare personnel on infection prevention protocols and proper device care. Enhanced surveillance programs to monitor device-related infections and identify trends or outbreaks promptly are crucial. Furthermore, optimizing antimicrobial stewardship programs to minimize unnecessary antibiotic use can help prevent the development of multidrug-resistant infections associated with these devices.

In conclusion, the COVID-19 pandemic has led to a predominance of infections caused by carbapenem-resistant bacteria, which present even more limited treatment options. In pandemic situations, strategies to control DAI should be maintained, as should factors predicting infections by resistant bacteria, such as length of stay in the ICU and duration of mechanical ventilation.

## Figures and Tables

**Table 1:** Characteristics of device-associated infections according to the diagnosis of COVID-19 in patients hospitalized in ICU.

Device-Associated Infections Characteristics	Total (n = 1665) No (%)	COVID-19 (n = 1143) No (%)	NO COVID-19 (n = 522) No (%)	*p* Value
Type VAP * CLABSI * CAUTI *	802 (48.2) 538 (32.3) 325 (19.5)	618 (54.1) 361 (31.6) 164 (14.3)	184 (35.2) 177 (33.9) 161 (30.8)	<0.001 0.347 <0.001
Two or more DAI	124 (8.1)	92 (8.9)	32 (6.6)	0.118
Time in days from hospitalization to the first DAI (Median (IQR *)	12 (8–17)	12 (8–16)	13 (9–20)	<0.001
Presence of devices Urinary catheter Central venous catheter Mechanical ventilation	1100 (66.1) 1137 (68.3) 1336 (80.2)	751 (65.7) 809 (70.8) 1007 (88.1)	349 (66.9) 328 (62.8) 329 (63.0)	0.645 0.001 <0.001
Days with the device Median (IQR *) Urinary catheter (n = 960) Mechanic ventilation (n = 985) Central catheter (n = 986)	13.5 (9–21) 15 (10–23) 13 (9–19)	14 (10–21) 15 (10–22) 13 (9–19)	12.5 (8–21) 15 (9–25) 12 (8–21)	0.025 0.601 0.767
Microorganisms (n = 989) *Staphylococcus aureus* *Enterococcus* spp. *Klebsiella pneumoniae* *Escherichia coli* *Enterobacter cloacae* *Pseudomonas aeruginosa* *Acinetobacter baumannii*	60 (6.1) 88 (8.9) 285 (28.9) 159 (16.1) 60 (6.1) 108 (10.9) 3 (0.3)	38 (6.3) 58 (9.6) 195 (32.2) 78 (12.9) 38 (6.3) 58 (9.6) 1 (0.2)	22 (5.8) 30 (7.8) 90 (23.6) 81 (21.2) 22 (5.8) 50 (13.1) 2 (0.5)	0.738 0.352 0.003 0.001 0.738 0.086 0.333
Polymicrobial infection	69 (6.7)	36 (5.9)	33 (8,6)	0.107

* VAP: ventilator-associated pneumonia; CAUTI: catheter-associated urinary tract infection; CLABSI: central-line-associated bloodstream infection; IQR: interquartile range.

**Table 2:** Predictors of infections by resistant bacteria in patients with COVID-19.

Outcome	Bivariate Analysis				Multivariate Analysis			
	RR	CI 95%		*p* Value	RR	CI 95%		*p* Value
Meticillin-resistant *Staphylococcus aureus*								
Age over 60 years	1.16	0.26	5.14	0.840	1.00	0.27	3.70	0.997
ICU stay > 14 days	0.65	0.10	4.25	0.651	0.65	0.10	4.07	0.644
Diabetes	1.10	0.50	2.41	0.813	1.27	0.62	2.58	0.514
Carbapenem-resistant *Enterobacteriaceae*								
Age over 60 years	1.80	0.96	3.38	0.065	1.42	0.77	2.65	0.264
ICU stay > 14 days	2.20	1.51	3.19	<0.001	2.09	1.39	3.16	<0.001
Diabetes	1.84	1.30	2.60	0.001	1.73	1.21	2.49	0.003
Mechanic ventilation	1.87	1.00	3.53	0.054	2.13	0.99	4.51	0.049
Central venous catheter	0.68	0.19	2.36	0.541	0.53	0.14	2.02	0.352
Carbapenem-resistant non-fermenting Gram-negative bacilli								
Age over 60 years	1.22	0.39	3.77	0.734	1.19	0.41	3.45	0.746
ICU stay > 14 days	2.59	0.99	6.78	0.053	2.47	0.95	6.44	0.065
Diabetes	0.82	0.26	2.55	0.732	0.79	0.26	2.43	0.681
Mechanic ventilation	1.74	0.54	5.63	0.355	1.55	0.29	8.30	0.607
Central venous catheter	1.38	0.31	6.17	0.675	1.05	0.15	7.42	0.964
ESBL-producing Gram-negative bacilli								
Age over 60 years	0.87	0.56	1.37	0.555	0.83	0.50	1.38	0.476
ICU stay > 14 days	1.06	0.55	2.03	0.857	1.07	0.54	2.12	0.854
Diabetes	1.13	0.68	1.87	0.644	1.18	0.70	1.96	0.537
Urinary catheter	1.48	0.61	3.60	0.383	1.51	0.61	3.74	0.375

## Data Availability

Data are contained within the article and Supplementary Materials.

## Supplementary Materials

The following supporting information can be downloaded at: https://www.mdpi.com/article/10.3390/microorganisms12040640/s1, Figure S1: Inclusion flowchart of patients with device-associated infections (DAI) hospitalized in ICU; Figure S2: Use of antimicrobials in defined daily dose (DDD) between March 2020 and May 2021 in intensive care units in Medellín; Table S1: Clinical and demographic characteristics of patients with device-associated infections according to COVID-19 diagnosis; Table S2: Isolated bacteria in patients with COVID-19, according to the type of DAI.
