# Supplementary material for: Device-Associated Infections in COVID-19 Patients: Frequency of Resistant Bacteria, Predictors and Mortality in Medellín, Colombia
Source: Microorganisms. 2024 Mar 22;12(4):640. doi: 10.3390/microorganisms12040640 (PMC11051839; doi:10.3390/microorganisms12040640)
Supplement: Supplementary file 1 [file microorganisms-12-00640-s001.zip › microorganisms-2872210-supplementary.pdf]

SUPPLEMENTARY MATERIAL

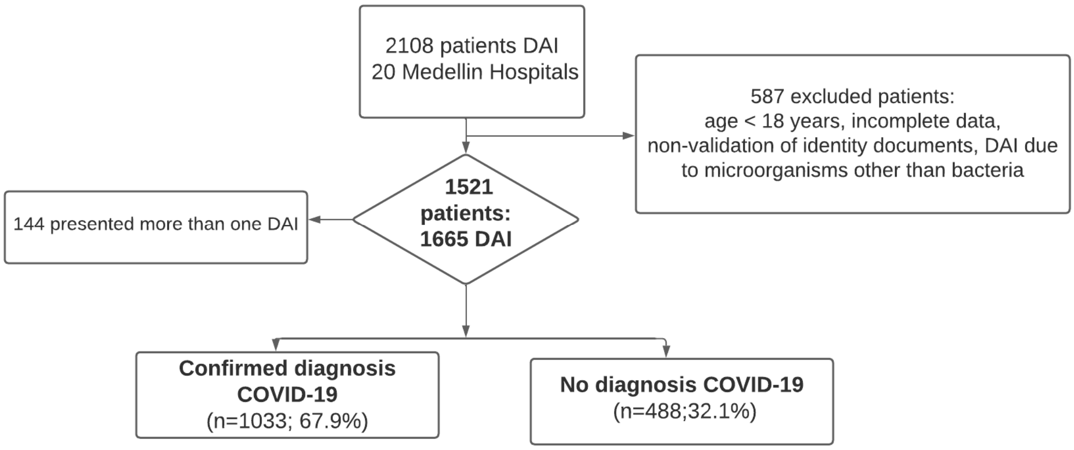

Figure S1. Inclusion flowchart of patients with device-associated infections (DAI) hospitalized in ICU. Medellín, March 2020–May 2021

Table S1. Clinical and demographic characteristics of patients with device-associated infections according to COVID-19 diagnosis

Patients characteristics	Total (n=1521) No (%)	COVID-19 (n=1033) No (%)	NO COVID-19 (n=488) No (%)
**Sex**			
Male	954 (62.7)	662 (64.1)	292 (59.8)
Female	567 (37.3)	371 (35.9)	196 (40.2)
**Age Median (IQR*)**	64 (52-71)	64 (54-71)	62 (48-70)
**Country of birth**			
Colombia	1502 (98.7)	1029 (99.6)	473 (96.9)
Venezuela	15 (1.0)	3 (0.3)	12 (2.5)
Peru	3 (0.2)	1 (0.1)	2 (0.4)
Brazil	1 (0.1)	0	1 (0.2)
**Referred patient**	344 (22.6)	230 (22.3)	114 (23.4)
**Comorbidities**			
Cancer	76 (5.0)	38 (3.7)	38 (7.8)
Malnutrition	15 (1.0)	7 (0.7)	8 (1.6)
Diabetes	336 (22.1)	237 (22.9)	99 (20.3)
Renal failure	91 (6.0)	54 (5.2)	37 (7.6)
COPD*	118 (7.8)	81 (7.8)	37 (7.6)
Immunosuppression	50 (3.3)	23 (2.2)	27 (5.5)
HIV-AIDS*	9 (0.6)	5 (0.5)	4 (0.8)

Previous infection	216 (14.2)	173 (16.7)	43 (8.8)
Trauma	19 (1.2)	6 (0.6)	13 (2.7)
Obesity	298 (19.6)	232 (22.5)	66 (13.5)

*IQR: Interquartile range, COPD: chronic obstructive pulmonary disease, HIV-AIDS: human immunodeficiency virus - acquired immunodeficiency syndrome

Table S2: Isolated bacteria in patients with COVID-19, according to the type of DAI

Bacteria	VAP*	CLABSI*	CAUTI*
Acinetobacter spp.	2	1	0
Alcaligenes xylosoxidans	0	1	0
Burkholderia spp.	0	9	0
Citrobacter spp.	1	5	2
Enterobacter spp.	7	31	14
Enterococcus spp.	1	34	20
Escherichia coli	3	15	60
Haemophilus influenzae	0	1	0
Hafnia alvei	0	2	1
Klebsiella spp.	0	1	5
Klebsiella oxytoca	1	7	2
Klebsiella pneumoniae	33	125	38
Morganella morganii	0	0	1
Pantoea spp.	0	1	0
Proteus mirabilis	0	3	9
Providencia rettgeri	1	0	0
Pseudomonas aeruginosa	15	4	18
Pseudomonas flurorescens	0	0	19
Serratia marcescens	4	25	2
Staphylococcus aureus	12	25	1

Coagulase-negative *Staphylococcus*	3	62	0
Stenotrophomonas maltophilia	0	3	0
Streptococcus spp.	0	4	1
**Total**	83	359	193

* Ventilator-associated pneumonia (VAP), central-line-associated bloodstream infection (CLABSI) and catheter-associated urinary tract infection (CAUTI).

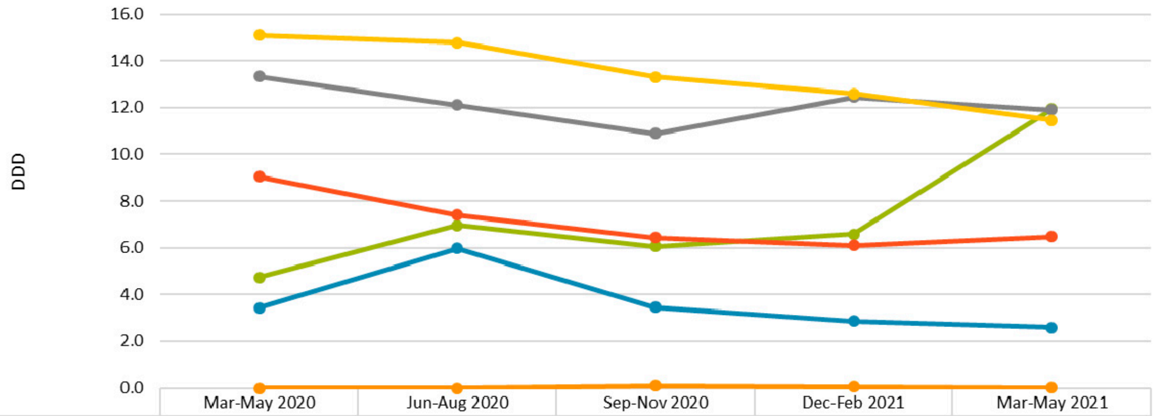

	Mar-May 2020	Jun-Aug 2020	Sep-Nov 2020	Dec-Feb 2021	Mar-May 2021
DDD Ceftriaxone	3.4	6.0	3.5	2.9	2.6
DDD Cefepime	4.7	7.0	6.1	6.6	12.0
DDD Ertapenem	0	0	0.1	0.06	0.02
DDD Meropenem	13.3	12.1	10.9	12.4	11.9
DDD Pip/Taz	15.1	14.8	13.3	12.6	11.5
DDD Vancomycin	9.0	7.4	6.4	6.1	6.5

DDD Ceftriaxone DDD Cefepime DDD Ertapenem DDD Meropenem DDD Pip/Taz DDD Vancomycin

Figure S2. Use of antimicrobials in Defined Daily Dose (DDD) between March 2020 and May 2021 in intensive care units in Medellín. Pip/Taz: Piperacillin-tazobactam
